# Characterization and Whole Genome Analysis of Human Papillomavirus Type 16 E1-1374^∧^63nt Variants

**DOI:** 10.1371/journal.pone.0041045

**Published:** 2012-07-24

**Authors:** Ivan Sabol, Mihaela Matovina, Ali Si-Mohamed, Magdalena Grce

**Affiliations:** 1 Division of Molecular Medicine, Rudjer Boskovic Institute, Zagreb, Croatia; 2 Department of Microbiology and Parasitology, School of Medicine, University of Rijeka, Rijeka, Croatia; 3 Laboratoire de Virologie, Hôpital Européen Georges Pompidou, Paris, France; 4 Division of Molecular Medicine, Rudjer Boskovic Institute, Zagreb, Croatia; National Institute of Health - National Cancer Institute, United States of America

## Abstract

**Background:**

The variation of the most common Human papillomavirus (HPV) type found in cervical cancer, the HPV16, has been extensively investigated in almost all viral genes. The E1 gene variation, however, has been rarely studied. The main objective of the present investigation was to analyze the variability of the E6 and E1 genes, focusing on the recently identified E1-1374^∧^63nt variant.

**Methodology/Principal Findings:**

Variation within the E6 of 786 HPV16 positive cervical samples was analyzed using high-resolution melting, while the E1-1374^∧^63nt duplication was assayed by PCR. Both techniques were supplemented with sequencing. The E1-1374^∧^63nt duplication was linked with the E-G350 and the E-C109/G350 variants. In comparison to the referent HPV16, the E1-1374^∧^63nt E-G350 variant was significantly associated with lower grade cervical lesions (p = 0.029), while the E1-1374^∧^63nt E-C109/G350 variant was equally distributed between high and low grade lesions. The E1-1374^∧^63nt variants were phylogenetically closest to E-G350 variant lineage (A2 sub-lineage based on full genome classification). The major differences between E1-1374^∧^63nt variants were within the LCR and the E6 region. On the other hand, changes within the E1 region were the major differences from the A2 sub-lineage, which has been historically but inconclusively associated with high grade cervical disease. Thus, the shared variations cannot explain the particular association of the E1-1374^∧^63nt variant with lower grade cervical lesions.

**Conclusions/Significance:**

The E1 region has been thus far considered to be well conserved among all HPVs and therefore uninteresting for variability studies. However, this study shows that the variations within the E1 region could possibly affect cervical disease, since the E1-1374^∧^63nt E-G350 variant is significantly associated with lower grade cervical lesions, in comparison to the A1 and A2 sub-lineage variants. Furthermore, it appears that the silent variation 109T>C of the E-C109/G350 variant might have a significant role in the viral life cycle and warrants further study.

## Introduction

Human papillomaviruses (HPV) are small, double stranded DNA viruses. Even though there exist more than 100 different genotypes, only about 40 infect the human anogenital tract. At least 13 oncogenic or high-risk HPV types are involved in the development of neoplasia and cancer, notably cervical cancer [1]. The HPV types themselves are subdivided into viral variants, which themselves have been shown to have differing oncogenic potential [2].

HPV16 is the most prevalent HPV type in cervical cancer cases worldwide [3] and is also the most prevalent HPV type in other lesions [4]. Many studies have focused on HPV16 variability in different regions of the HPV genome, mostly the E6 and E7 oncogenes [5]. The variability of the E2 and late genes, L1 and L2, along with the long control region (LCR), has also been analyzed [6]. Studies focusing on E1 region, however, are limited. Previously we found a 63-nucleotide duplication, at position 1374 within the E1 gene (E1-1374^∧^63nt), in about 10% of HPV16 positive cervical samples. This finding indicates that this particular variation is relatively common in the Croatian population [7], and possibly elsewhere. The same variant was also confirmed to be present in neighbouring Slovenia, in about 8% of samples [8]. The HPV16 variant containing this duplication was more strongly linked with low-grade cervical lesions than the reference HPV16 (E–r; European prototype) [7]. Furthermore, all samples containing the E1-1374^∧^63nt duplication belonged to the commonly reported E-G350 (E6-350G or L83V) variants [7]. The E-G350 variant is still controversial in terms of its oncogenicity, which has been found to vary significantly across different studies [5].

The first goal of this study was to analyze the variability of the E6 gene together with the E1 gene, focusing on the recently identified E1-1374^∧^63nt variant. The second objective was to evaluate the association of the E1-1374^∧^63nt variant with different grades of cervical lesions on a large number samples in order to clarify the association of this variant with lower grade cervical lesions. In this study, we focused on E6 region to eliminate the possibility that the previously known HPV16 E6 variants affect this association. The final goal was to determine if nucleotide changes in other regions of the HPV16 genome might be responsible for the observed association. For this reason we sequenced the whole genome of this E1 variant.

## Materials and Methods

### DNA Collection

In this study, 786 individual HPV16 positive archival DNA samples of cervical smears collected and stored in the local DNA Biobank from 1999 to 2009 at the Division of Molecular Medicine (Rudjer Boskovic Institute, Zagreb, Croatia) were analyzed. HPV detection and genotyping was done with two consensus primer sets, MY09/11 (up to 2006, PGMY09/11 thereafter) and L1C1, and type-specific primers for HPV types 6/11, 16, 18, 31, 33, 45, 52 and 58, as previously described by Milutin-Gasperov et al. [9]. When available, patient cytological diagnosis was used for statistical analysis [10]. Average age of examinees was 29.1 (11-69).

### Ethics Statement

Verbal patient consent was obtained for each cervical specimen that was collected for HPV diagnostic and research purposes. Direct written consent was not required. Namely, the cervical sample collection is regulated through Laboratory service request forms, which have to be signed and approved by the practicing physician. These forms serve to document the indirect consent of each patient. Both the DNA extracted from cervical specimens and the relevant patient data (age, cytological diagnosis, HPV detection and typing result), were processed anonymously. The study was funded by the Croatian Ministry of Science, Education and Sport, through Research Grant No. 098-0982464-2510, and approved by the Ethical Board of the Rudjer Boskovic Institute (Zagreb) as well as the Ethical Board of the "Sisters of Mercy" Hospital (Zagreb) and is in line with the Helsinki declaration (DoH/Oct2008).

### E6 Variants Analysis

The high resolution melting (HRM) method was used to determine HPV16 variability, as described previously [11]. Briefly, primers designed by Ortiz et al. [12] and Sotlar et al. [13] were adapted to be used in the nested polymerase chain reaction (PCR) [11]. The inner PCR was performed in the presence of the double-stranded-DNA-binding fluorescent dye LCgreen+ and the fluorescence emission during the sample heating was recorded on the HR1 instrument (Idaho Technologies, USA). The resulting melting curves were compared to the melting curves of simultaneously run reference samples within the HR1 software and the deviations indicated E6 region variants.

### E6 Variant Sequencing

Samples with melting curves that differed from the reference were sequenced after amplicon purification using Promega Wizard(R) SV Gel and PCR Clean-Up System (Promega, USA). Ten randomly selected reference (E-r) and ten randomly selected E-G350 variants were sequenced for assay verification. Samples were sequenced using both forward and reverse primers on the 3730xl DNA Analyzer (Applied Biosystems, USA) at the European Hospital Georges Pompidou, Paris, France.

### E1 Duplication Analysis

The E1-1374^∧^63nt duplication was detected using specific primer directed PCR for the E1 region, as described previously [14]. Briefly, primers that amplify 189 bp of the E1 region were used. If the duplication is present then the E1 amplicon is shifted to around 250 bp.

### Whole Genome Sequencing

The HPV genome of two randomly selected samples of the E1-1374^∧^63nt E-G350 variant and two additional randomly selected samples of the E1-1374^∧^63nt E-C109/G350 variant were completely sequenced using primers and conditions from the study by Bhattacharjee et al. [15] with additional modifications. Thus, several additional primer pairs were used for the sequencing of the E1 region: E1-A1 (5'-CTCAGAAACCATAATCTACC) with E1–B2 (5'-CTAATAGTAACACAACCATTCC), E1–A2 (5'-CAAAGTTTAGCATGTTCATGG) with E1–B4 (5'-GTAGCATCATCTAACATACC), E1–A3 (5'-CACAGGCAAAAATTGTAAAGG) with E1–B5 (5'-GTCTATATGGTCACGTAGG), and E1-A4 (5'-GTTAGATGATGCTACAGTGCC) with E1–B5 primer. Cycling parameters consisted of initial denaturation for 5 minutes at 95°C, 40 cycles of 30 seconds of denaturation at 95°C, 45 seconds of annealing at 55°C, 1 minute of elongation at 72°C with final elongation of 7 minutes at 72^°^C. The primers L1–2, from Bhattacharjee et al. [15] were replaced with Alt16L1-F (5'-AGGTCGTGGTCAGCCATTAG) and Alt16L1-R (5'-GGGGATCTTCTTTAGGTGCTG). Cycling parameters were: initial denaturation for 2 minutes at 95°C, 35 cycles of 20 seconds of denaturation at 95°C, 20 seconds of annealing at 63°C, and 50 seconds elongation at 72°C with final elongation of 7 minutes at 72°C. All PCR reactions (50µl) contained GOTaq-green-PCR-buffer (Promega), 3000µM MgCl_2_, 100µM each dNTP, 0.2µM of each primer, 50ng sample DNA and 1U GOTaq-polymerase (Promega). Amplicons were purified using Promega system as above and sequenced using forward and reverse primers at the ABI PRISM 310 Genetic Analyzer (Applied Biosystems, USA) at the Rudjer Boskovic Institute (Zagreb, Croatia) core facility.

Amplicon sequences were aligned with the reference sequence using Bioedit version 7.0.5.2. Continuous sequences of variant samples ZG01-118 and ZG01-258 were created for the E1-1374^∧^63nt E-G350 variant. Continuous sequences of variant samples ZG03-145 and ZG05-249 were created for the E1-1374^∧^63nt E-C109/G350 variant.

### E1 Region Sequencing

The same primers that were used for the whole genome sequencing of the E1 region (above) were used to sequence the E1 region of 10 additional E1-1374^∧^63nt E-G350 variants and 4 additional E1-1374^∧^63nt E-C109/G350 variants.

### Nomenclature of Variants and Reference HPV16 Genome

The particular variant names used in this study were chosen according to the proposed HPV16 variant nomenclature [16]. Briefly, variants are named according to their lineage (E, AA, As, NA1, Af1, Af2) followed by the variant class (only for non European variants) and the subclass that corresponds to nucleotide(s) present at specified position(s) that are different from the respective reference sequence; i.e. E-G350 corresponds to the European lineage variant with G at position 350. The HPV16R reference sequence was obtained from Papillomavirus Episteme, a specialized information database for Papillomaviridae family of viruses (http://pave.niaid.nih.gov/). Other variant reference sequences were obtained from NCBI GenBank database (http://www.ncbi.nlm.nih.gov/): AA variant (AF402678), Af1 (AF536180) and Af2 (AF472509). Sequences and alignment of previously published whole genomes of HPV16 variants [17] were kindly provided by Prof R.D. Burk.

A classification scheme based on full genome analysis has been recently proposed [17]–[19]. Using the same approach, we have classified the variants sequenced in this study to assess to which lineage or sub lineage they belong (according to their full genome sequence) and compared them with the previously published whole genome sequences [17]. The MEGA version 5.05 [20] software package was used to create maximum likelihood trees and to calculate pairwise nucleotide sequence differences between each isolate and all others.

### Structural Prediction of the E1 Protein

The three dimensional structures of the variant and the reference E1 protein were determined from the respective full length amino acid sequences with three different structure prediction web servers, SAM-T08 [21], I-TASSER [22] and Phyre2 [23], based on automated homology modelling. The resulting models were visualized and analyzed using the Chimera software [24]. The most likely predicted models were compared to and superimposed over the already solved partial structures of the E1 protein deposited in the protein data bank (PDB) under PDB IDs: 2GXA [25], 1TUE [26] and 1F08 [27]. The I-TASSER server has additionally been used to optimize the E1 referent predicted structure using the Phyre2 E1 variant model as a constraint.

### Statistical Analysis

The standard Chi-square (χ^2^) test was used to study associations between two variables and was calculated using GraphPad Prism (version 4.00) (GraphPad Software, San Diego, California, USA). The significance level was set to p<0.05.

## Results

The HRM analysis of the E6 amplicon was successful in 722 of 786 (91.9%) individual samples. The European lineage G350 variant (E-G350) was identified by HRM analysis in 358 (51.4%) cases, while those of the European prototype T350 (E-r) were found to occur in 212 (30.4%) cases. Two samples had melting profiles of a mixed infection of E-G350 and E-r variants. In addition, 150 samples had discrepant or completely different melting profiles of the E6 region and were, therefore, sequenced to identify the exact variants. There were 16 samples positive for the E6 analysis that did not give discernible E1 amplicons. These samples, which probably have partial disruptions of the E1 region due to integration or other rearrangement events, were excluded from further analysis.

As the HRM method was previously found to be accurate for viral variant detection [11], only twenty more randomly selected E-G350 and E-r samples (10 each), chosen as control samples, were sequenced. Both strands of DNA were sequenced to avoid sequencing artefacts and such sequencing of the control samples confirmed the HRM findings in each case (data not shown). The resulting sequences were grouped and named according to the proposed nomenclature [16]. Variants that were found in less than 5 samples each, were grouped with the most similar variants and suffixed with "other" to indicate the presence of other nucleotide changes (E-r other, E-G350 other, NA1 other) (Table 1). The exact sequences of 126 samples that contained variations in the E6, other than the most common G350 variation, are presented in Supplement material (Table S1). The total prevalence of the E-G350 variant, including samples with discrepant HRM findings that were subsequently sequenced, was 52.1% (376/722), while E-r was found in 31.2% (225/722) samples.

**Table 1:** Distribution of the HPV16 variants within different grades of cervical lesions.

			Patient cytological diagnosis a				
E1 status	Variant group b	No. c	U	NC	ASCUS	LSIL (%)	HSIL (%)
E1-1374^∧^63nt variant	E-G350	46	3	0	9	16 (34.8%)	18 (39.1%)
	E-G350 other	5	0	0	1	2 (40%)	2 (40%)
	E-C109/G350	22	2	0	3	4 (18.2%)	13 (59.1%)
Subtotal E1 variant		73	5	0	13	22 (30.1%)	33 (45.2%)
E1 reference	E-r	220	18	1	36	45 (20.5%)	120 (54.5%)
	E-r other	20	1	0	5	5 (25%)	9 (45%)
	E-G350	319	29	2	56	67 (21%)	165 (51.7%)
	E-G350 other	39	2	1	6	5 (12.8%)	25 (64.1%)
	E-C109/G350	9	1	0	3	0 (0%)	5 (55.6%)
	E-G131/G350	6	1	0	0	1 (16.7%)	4 (66.7%)
	E-G350+ E-r	3	0	0	0	0 (0%)	3 (100%)
	Subtotal E	616	52	4	106	123 (20%)	331 (53.7%)
	NA1-b/r	8	1	0	1	3 (37.5%)	3 (37.5%)
	NA1 other	2	0	0	0	0 (0%)	2 (100%)
	AA-a/r	2	0	0	1	1 (50%)	0 (0%)
	Af1-b/r	3	0	0	2	0 (0%)	1 (33.3%)
	Af2-a/C109/G403	1	1	0	0	0 (0%)	0 (0%)
	Subtotal Non-E	16	2	0	4	4 (25%)	6 (37.5%)
Subtotal E1 reference		632	54	4	110	127 (20.1%)	337 (53.3%)
Total		705	59	4	123	149 (21.1%)	370 (52.5%)

a U, unknown diagnosis; NC, normal cytology; ASCUS, atypical squamous cells of undetermined significance; LSIL, low grade squamous intraepithelial neoplasia; HSIL, high grade squamous intraepithelial neoplasia.

b variant names according to the recently proposed nomenclature [16]; E-G350 other, all other variants containing 350G and other variations but found in less than 5 samples each; E-r other, all other variants containing 350T with other variations.

c No., number of samples.

d this was statistically significant in comparison to E1 reference E-r (p = 0.0227), E1 reference E-G350 (p = 0.0326), E1 reference subtotal E (p = 0.0128) and subtotal E1 reference samples (p = 0.0143).

e this was statistically significant in comparison to E1 reference subtotal E (p = 0.0452) and borderline significant in comparison with subtotal E1 reference samples (p = 0.0503).

The E1 amplification was successful in 736 samples (93.6%). There were 30 samples positive for the E1 analysis (PCR amplicon size 189), which were also repeatedly negative for the E6 analysis (HRM amplicon size 523 bp), probably due to the degradation of the DNA. Those samples were excluded from further analysis as well as one sample containing both referent and variant E1 amplicon.

The combined findings, covering 705 individual samples with successful E1 and E6 analysis complemented with patient cytological diagnosis, are presented in Table 1. The E1-1374^∧^63nt duplication was found in only two abundant variant groups, E-G350 and E-C109/G350, and in 5 other sporadic variants related to the E-G350 variant (E-G310/G350, E-T91/G350/C432, E-G350/C473, E-C176/G350, E-T246/G350). The common feature of all variant groups with the E1-1374^∧^63nt duplication is that they are related to the E-G350 variant. Even though a large number of samples was examined, all samples contained both the G350 change and the E1-1374^∧^63nt duplication; this exclusive association was highly significant (χ^2^ = 43.64, p<0.0001). In addition, the E1-1374^∧^63nt duplication was seen only in the European lineage variants. This result might be influenced by the low number of non-European variants detected in the Croatian population.

The frequency of high and low grade lesions in different variant groups and subgroups was examined and compared to the reference variant (Table 1). Of all the samples in which the reference variant was found, more than half were diagnosed as HSIL (120/220; 54.5%) and only 20.5% as LSIL (45/220). Similar frequencies were found for the E-G350 variant, 51.7% as HSIL (165/319) and 21% as LSIL (67/319) cases. In contrast, the E1-1374^∧^63nt E-G350 variant (the most common variant with the E1-1374^∧^63nt duplication found in this study) was associated almost equally with LSIL (16/46; 34.8%) and HSIL (18/46; 39.1%) diagnosis; this association was significant in comparison with several variant groups that could be considered as a good reference for comparison (Table 1). Thus, the E1-1374^∧^63nt E-G350 variant was found to be significantly more associated with lower grade lesions than the E-r variant (χ^2^ = 5.192, p = 0.0227), the E-G350 variant without the E1-1374^∧^63nt duplication (χ^2^ = 4.565, p = 0.0326), all European lineage samples without the duplication (χ^2^ = 6.190, p = 0.0128) and all samples without the duplication (χ^2^ = 5.999, p = 0.0143). The only significant comparison of all E1-1374^∧^63nt variants (Table 1, subtotal E1 variant) were those with all European lineage samples without the duplication (Table 1, subtotal E [European]; χ^2^ = 4.012, p = 0.0452), while the comparison with all E1 variants without the duplication was at the borderline of statistical significance (Table 1, subtotal E1 reference; χ^2^ = 3.832, p = 0.0503).

**Figure 1:**
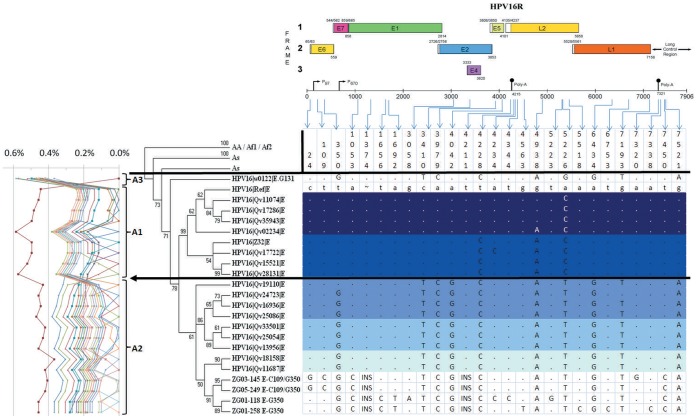
Phylogenic clustering of E1-1374^∧^63nt variant sequences with other currently available published HPV16 whole genome sequences. The whole genome sequences of four E1-1374^∧^63nt variant samples (ZG01-118, ZG01-258, ZG03-145 and ZG05-249) were aligned with the available published whole genome sequences and the phylogenetic tree was created using MEGA5. The leftmost part shows the percent nucleotide sequence differences calculated using MEGA5 to determine variant lineages according to a proposed taxonomic classification [18], [19]. The top part of the figure was adapted from the transcription map of HPV16 available at http://pave.niaid.nih.gov/. Sequences at respective positions are shaded in different shades of blue according to the distance from the E1-1374^∧^63nt variant. Dots "." indicate no change from referent sequence while "∼" and "INS" indicate insertions. The European (or A) lineage appears to be subdivided into 3 sublineages A1–A3, based on the tree topology and sequence percent difference similarly as previously described [17]–[19]. The A1 lineage contains the reference HPV16 genome, the A2 lineage contains the E-G350 related variants, including the E1-1374^∧^63nt variant samples and the E-12 variant described in Bhattacharjee et al. [15]. The A3 sublineage contains only the E-G131 sample, which exhibits the most difference from other sequences. Variant "E-12" was previously shown to be more prevalent in cancer samples [15], while the variation 7450 T>C was shown to be statistically more prevalent in cancer samples [49]. Positions where samples ZG01-118 and ZG01-258 differ have been sequenced for 3 additional samples and in each case only one of 5 sequenced samples has this change. Additional whole length E1 sequencing has revealed that changes at positions 1656 and 1692 were only present in 2 of 12 samples. Thus, all those changes are unlikely to be the major cause of the association of the E1-1374^∧^63nt E-G350 variant with low grade cervical lesions. The major difference between E1-1374^∧^63nt E-G350 variant and other variants are the positions 1053 and 1374, while the only differences between the E-G350 and E-C109/G350 sequenced variants are at positions 24 and 109.

Sequencing of the whole genome of two samples with the E1-1374^∧^63nt E-G350 variant revealed a total of 16 common changes (Table 2; Figure 1). Combined sequences of samples ZG01-118 and ZG01-258 were submitted to the NCBI GenBank and assigned accession numbers, JN565302 and JN565303, respectively. Combined E1 and E6 analysis has revealed that E1-1374^∧^63nt duplication is also present in the E-C109/G350 variant. Thus, the whole genomes from two randomly selected samples of E1-1374^∧^63nt E-C109/G350 variant were sequenced, as well. Combined whole genome sequences of samples ZG03-145 and ZG05-249 were submitted to the NCBI GenBank and assigned accession numbers, JQ067943 and JQ067944, respectively (Table 2; Figure 1).

**Table 2:** Positions of nucleotide and amino acid changes within the whole genome and E1 sequences of the major HPV16 variants containing the E1-1374^∧^63 duplication.

Curated referent sequence position a	Referent sequence	Nucleotide changes (number observed/number sequenced) b		Description of the variation
		E1-1374^∧^63 E-G350	E1-1374^∧^63 E-C109/G350	
24	C	C (2/2)	**G (2/2)** c	Silent
109	T	T (2/2)	**C (2/2)** c	E6 silent
350	T	**G (2/2)**	**G (2/2)**	E6 83 L > V d
**1053**	**A**	**C (12/12)**	**C (6/6)**	**E1 63 E > D** d
**1374ins**	**-**	**INS (12/12)**	**INS (6/6)**	**E1 duplication of 63 nucleotides**
**1656**	**T**	**C (2/12)**	**T (6/6)**	**E1 silent**
**1692**	**A**	**T (2/12)**	**A (6/6)**	**E1 276 (297) L > F**
**2184**	**G**	**A (1/12)**	**G (6/6)**	**E1**
3058	G	**A (1/5)**	G (2/2)	E2 silent
3410	C	**T (2/2)**	**T (2/2)**	E2 219 P>S and E4 silent d
3979	A	**C (2/2)**	**C (2/2)**	E5 44 I > L d
4042	A	**G (2/2)**	**G (2/2)**	E5 65 I > V d
4211 ins	-	**GTTT (2/2)**	**GTTT (2/2)**	Silent Insertion of GTTT d
4211 ins	-	**GTT (1/5)**	- (2/2)	Silent insertion of GTT
4228	T	**C (2/2)**	**C (2/2)**	Silent d
4234	A	**C (1/5)**	A (2/2)	Silent
**4344**	**T**	**C (1/5)**	T (2/2)	**L2 silent**
4563	G	**T (1/5)**	G (2/2)	L2 silent
4938	G	**A (2/2)**	**A (2/2)**	L2 silent d
**5223**	**T**	**G (1/5)**	T (2/2)	**L2 329 D>E**
5226	A	**T (2/2)**	**T (2/2)**	L2 330 L>F d
5518	A	**C (1/5)**	A (2/2)	L2 428 I>L
6434	A	**G (2/2)**	**G (2/2)**	L1 292 T>A d
**6753**	**T**	**C (1/5)**	T (2/2)	**L2 398 L>S**
7193	G	**T (2/2)**	**T (2/2)**	Silent d
7320	A	A (2/2)	**G (1/2)**	Silent
**7338**	**A**	**C (1/5)**	A (2/2)	**Silent**
7450	T	**C (2/2)**	**C (2/2)**	Silent d
7521	G	**A (2/2)**	**A (2/2)**	Silent d

a novel nucleotide changes are highlighted in bold and underlined.

b changes from the reference are highlighted in bold.

c variations differentiating E1-1374^∧^63nt duplication containing E-G350 and E-C109/G350 variants.

d variation present in all samples.

The full length E1 region sequencing was done on 10 additional E1-1374^∧^63nt E-G350 and 4 additional E1-1374^∧^63nt E-C109/G350 variant samples, and the results are also presented in Table 2. There was only one additional change within the E1 region (2184 G>A) that was not seen in the full genome sequences.

Throughout the rest of the whole genome of the E1-1374^∧^63nt E-G350 variant there were 9 additional sites where only a single sample had a specific nucleotide change. In these cases, 3 more samples of the E1-1374^∧^63nt E-G350 variant were sequenced to clarify each of these particular regions and derive the consensus sequence. In all such cases, only one of 5 completely sequenced samples for each region had the variation, while the other four had sequences identical to the reference sequence.

The sequences of the E1-1374^∧^63nt variant samples were compared to the previously published HPV16 variant sequences [17] and notable differences are presented in Figure 1. There are 10 nucleotide changes that are present in both E1-1374^∧^63nt duplication containing variants and the phylogenetically closest variants (A2 sub-lineage), while E1-1374^∧^63nt E-G350 and E1-1374^∧^63nt E-C109/G350 share 14 identical variations. The only differences between E1-1374^∧^63nt E-G350 and E1-1374^∧^63nt E-C109/G350 variants were LCR-G24, E6-C109, E1-C1656 and E1-T1692. The LCR-G24 and E6-C109 were specific for the E1-1374^∧^63nt E-C109/G350 variant. The E1-C1656 and E1-T1692 were specific for the E1-1374^∧^63nt E-G350 variant, but they were found only in 2 of 12 full length E1 sequences of this variant.

The E1-1374^∧^63nt E-G350 variant had only a few changes in the known oncogenes (Tables 2, Figure 1). In the E6 region there was only one missense variation, the G350 (L83V) variation, while E7 region was completely free of variation. However, there were two missense variations in the E5 oncogene I44L and I65V. The E4-region was also found to be free of amino acid changes, while E2, L1 and L2 each had one missense variation common to all sequenced E1-1374^∧^63nt E-G350 variant samples. The sequencing of the whole genome and additional full length E1 sequences revealed that all analyzed samples of the E1-1374^∧^63nt E-G350 variant had the E1 1053 A>C change and the E1-1374^∧^63nt duplication resulting with the insertion of 21 extra amino acids in the E1 protein. In addition, there were two silent and one missense variations, which were found in only a subset of the analyzed samples.

Phylogenetic analysis has shown that the pairwise difference in the nucleotide sequences is mostly under 0.5% between all the European-lineage samples compared. The European (or A) lineage was further subdivided into 3 sublineages (A1-A3) based on the tree topology and the sequence percent difference, in a manner similar to that described previously [17]–[19]. The A1 lineage contained the reference HPV16 genome, the A2 lineage contained the E-G350 related variants including the E1-1374^∧^63nt variants and the A3 lineage contained only the E-G131 variant that differed the most from other sequences (Figure 1). Due to the historical significance of the E-G350 variant, the most investigated member of the A2 sub-lineage (Figure 1), we have decided to use the tree topology approach in addition to sequence percent difference in order to better distinguish the E-G350 from the E-r variants. A similar approach was recently implemented for HPV11 variants [19].

**Figure 2:**
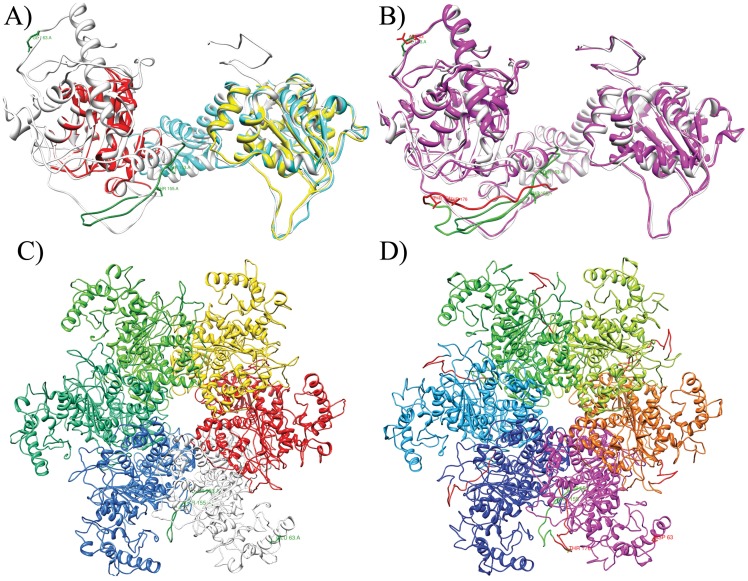
Prediction of E1 protein structure. Panel A shows 3D structure comparison between superposed monomer of the previously solved BPV1 E1 helicase domain hexamer structure (cyan; PDB ID:2GXA) [25], HPV18 E1 helicase domain structure (yellow; PDB ID:1TUE) [26], BPV1 E1 DNA binding domain structure (red; PDB ID:1F08) [27] and the predicted referent E1 model (white). The sequence at the position where the duplication occurs within the E1-1374^∧^63nt variants is highlighted in green, as is the amino acid 63 that is also changed in the E1-1374^∧^63nt variants. Panel B depicts superposed structures of the referent (white) and E1-1374^∧^63nt variant (magenta) E1 models. As before the referent sequence is highlighted in green and the changes specific to E1-1374^∧^63nt variant are highlighted in red. It can be seen that the newly added 21 amino acids (highlighted in red) partially overlap the potentially phosphorylated threonines (highlighted in green) at positions 153 and 155 and possibly influence the phosphorylation regulation of the E1 function. Panels C and D show the hexamer structure derived from the predicted E1 reference or E1-1374^∧^63nt variant proteins, respectively. The individual monomers are colored differently. The original referent sequence is colored in green within the white referent E1 monomer and the E1 variant magenta monomer. The sequence duplication in the E1-1374^∧^63nt variants is located at the junction between 2 adjacent E1 monomers within the structure and is highlighted in red on panel D. Positions of potentially phosphorylated threonines and amino acid substitution at position 63 are only highlighted on the white and magenta monomers.

Structural prediction of the variant and the referent E1 proteins using the SAM-T08 [21], I-TASSER [22] and Phyre2 [23] services resulted in several models. However, only the E1 Phyre2 model of the E1-1374^∧^63nt variant could be reconciled with the solved structure of the E1 hexamer (PDB ID:2GXA) [25], without any overlaps between individual E1 monomer structures. The E1 reference protein model was refined by the I-TASSER server using the Phyre2 E1-1374^∧^63nt variant structure as a constraint. Figure 2 presents the predicted full length structural models of the reference and the E1-1374^∧^63nt variant proteins. From the structural prediction, it appears that both E1-C1053 and E1-1374^∧^63nt are positioned on the surface of the E1 protein. Additional three dimensional representations of the predicted models are presented in Supplement material (Figure S1).

## Discussion

In the current study, we have focused on the interesting observation that the E1-1374^∧^63nt variant was unusually common and, more importantly, that it was significantly associated with low grade lesions [7]. To confirm that this variant is indeed very prevalent in the Croatian population, we have enlarged the sample pool to include 822 HPV16 positive cervical specimens. The prevalence of the E1-1374^∧^63nt variant remained about 10%, as previously determined on a subset of samples [7]. In addition, to resolve the observed association of the E1-1374^∧^63nt variant with low grade lesions, we had to eliminate the possible influence of other known variants. For that purpose we have analyzed the variant status of the E6 oncogene region, which is commonly analyzed in HPV variability studies. Sequencing of the samples that were preselected by the HRM clarified the variant status, with 20.8% of samples exhibiting melting curves that differed from the most common E-G350 and E-r curves. It is important to emphasize that the HRM analysis was employed only as a pre-screening tool to avoid repeated sequencing of the wild type or any other clearly distinguishable and common variant (E-G350 and E-r). All other samples were sequenced. In this regard, the HRM proved to be a powerful pre-screening method. Namely, of the 20.8% of samples subjected to sequencing, 79.2% were indeed identified as new variants. This approach enabled us to conduct one of the largest studies on HPV16 E6 variants so far.

Comparing the status of the E1-1374^∧^63nt duplication and different groups of E6 variants revealed that it is exclusively present in the E-G350 and the related variants. Around two thirds (63%) of all E1-1374^∧^63nt cases belonged to the E-G350 variant, while almost another third (30.1%) belonged to the E-C109/G350 variant (Table 1).

The hypothesis based on our previous study [7], that the originally identified E1-1374^∧^63nt E-G350 variant is more associated with LSIL lesions than the referent HPV16 variant, is further supported by data acquired on the substantially larger number of samples analyzed in the current study. In all comparisons with other relevant variant groups, the E1-1374^∧^63nt E-G350 variant was statistically significantly less associated with HSIL and more with LSIL (Table 1).

However, the variant E-C109/G350 with the E1-1374^∧^63nt duplication, identified in this study, is not in line with the previous hypothesis. Unexpectedly, this variant was more associated with HSIL diagnoses (59.1%) than even the reference (E-r) variant (54.5%) (Table 1). However, this observation might be influenced by the relatively small number of samples, as there were only 23 E1-1374^∧^63nt E-C109/G350 variant samples, and only 9 E1 referent E-C109/G350 variant samples. The E-C109/G350 variant itself was reported in many previous studies but, in almost every study, this variant was less prevalent than in the current sample pool, often being found in only 1 or 2 cases [6], [12], [28]–[33]. Exceptions can be found in the studies by Wheeler et al. [34] (8 of 67) and Zuna et al. [35] (18 of 354). Even though the E-C109/G350 variant appears to be rare in most of the populations studied and thus without major clinical significance, it has been noted in more than 20 publications worldwide which is a comparable to AA-a- r, (23 papers), NA-b-r (21 papers) and E-G131/G350 (26 papers), according to the recent comprehensive review on HPV16 variants [16]. This widespread presence suggests biological relevance of this variant, even if it was detected at low prevalence within most studies.

The full length E1 sequences and the whole genome sequences of HPV16 E1-1374^∧^63nt duplication containing E-G350 and E-C109/G350 variants revealed a total of 14 deviations from the reference HPV16 sequence, common for both variants, and 2 additional changes that differentiated the E-G350 and E-C109/G350 variants (indicated in Table 2 with superscript letters ^d^ and ^c^, respectively, and Figure 1). Variations at positions 1656 and 1692 were only detected in two whole genome sequences and not in 10 additional full E1 sequences and are thus considered not significant. There were 13 additional deviations found throughout the genomes that were present only in a subset of sequenced samples (Table 2).

Of all changes from the reference found in the HPV16 E1-1374^∧^63nt variant, the 350 T>G change has been reported most often. Some studies linked 350 T>G to higher oncogenic potential or viral persistence [36]–[41], but others did not [30], [33], [35], [42]–[44]. In addition, recent *in vitro* functional studies indicated advantages of this E-G350 variant over the reference HPV16 [45], [46], while another study found no difference between them [47]. In the current study, the E-G350 variant was slightly less associated with HSIL (51.7%) than the reference variant (54.5%). However, each of the E-G350 related variants (E-C109/G350, E-G350 other and E-G131/G350) were more associated to HSIL (55.6%, 64.1% and 66.7%, respectively) than the reference variant (Table 2). In either case, the consistent presence of change 350 T>G in the E1-1374^∧^63nt E-G350 variant is unlikely to be able to significantly decrease the association with HSIL of this variant or increase it with LSIL.

There have been many studies analyzing parts of the HPV16 genomes, and listing them all would exceed the scope of this paper. However, the majority of variations of the whole genome found within the E1-1374^∧^63nt variants in this study were also found in four studies that sequenced the whole genomes of 7 [32], 12 [48], 62 [17] and 98 samples [15]. These four studies detected 20 of 29 variations found in the E1-1374^∧^63nt variant and the majority of variations were observed in all 4 studies. The E1-1374^∧^63nt E-G350 variant samples have a similar profile to the 9 phylogenetically closest sequences, which exhibit changes from the reference at positions 350, 3410, 3979, 4042, 4228, 4938, 5226, 6434, 7193 and 7521 (Figure 1). This combination of variations, with an additional change at the position 7450, which is also specific for the E1-1374^∧^63nt variant, was previously described by Bhattacharjee et al. [15] to be the most prevalent variant in the Indian population and this specific variant was named E-12. More interesting is the report that the change 7450 T>C, within the LCR, is associated with cervical cancer [49]. The Indian variant E-12, that contains 7450 T>C change, was more prevalent in cancer cases than controls (38.2% vs. 28.5%); however, this was not statistically significant [15]. In any case, the Indian variant E-12 was more prevalent in cancer, and the 7450 T>C change significantly more so, making it unlikely that any of those changes (350, 3410, 3979, 4042, 4228, 4938, 5226, 6434, 7193, 7521 and 7450) could be responsible for the association of the E1-1374^∧^63nt E-G350 variant with lower grade lesions.

Within the whole genome, only 9 more novel changes in the E1-1374^∧^63nt E-G350 variant were identified (Table 2). Of those, 7 are found in a small subset of tested samples, making them unlikely to be responsible for the observed association with lower grade cervical lesions. The remaining 2 changes are the E1-1374^∧^63nt duplication itself and the 1053 A>C change, which leads to the substitution 63 E>D in the E1 protein. This change, along with the E1-1374^∧^63nt duplication, might affect the H1 histone, the DNA binding, the E1 protein oligomerization [50] or the regulation of the E1-E2 interaction [51]. Most of the functional studies on E1 were done on the BPV1 and HPV11 E1 proteins but, according to the review by Sverdrup and Myers [52], the E1 protein appears to be well conserved in sequence, structure and function. Lentz et al. [53] found Tyr126 within BPV E1 to be phosphorylated, while sequence alignment by Sverdrup and Myers [52] revealed no tyrosine at position 126 within the HPV16 E1. However, two tyrosine residues were found at 5 and 7 amino acid positions downstream, exactly within the region that is duplicated in the E1-1374^∧^63nt variants. Thus, we can speculate that the duplication might influence the phosphorylation of the HPV16 E1 protein as well, especially because it is known that the N-terminal part of the protein is involved in the regulation of the E1 function [50]. Furthermore, from the predicted structural model of the E1 proteins (Figure 2), it appears that this position is on the surface of the protein, making it accessible to phosphorylation. However, within the E1-1374^∧^63nt variants, the newly added 21 amino acids overlap the positions of potentially phosphorylated tyrosines (Figure 2D) making them probably less accessible. The newly added tyrosines are situated at almost the opposite part of the same loop (Figure 2D, coloured in red) and might not be phosphorylated or their phosphorylation might not have the same effect on the E1 protein. In addition, we can speculate that the gain of these 21 amino acids might sterically hinder oligomerization of the E1 protein or alter other protein interactions. The change 63 E>D (glutamate to aspartate) was also predicted to be situated at the surface of the protein (Figure 2B, 2D) and might also be involved in E1 protein-protein interactions. However, any influence the E1-1374^∧^63nt duplication might have does not seem to have a drastic effect on the pathogenicity of this HPV16 variant. Namely, the duplication is still found in HSIL lesions, albeit less often. In this light, it is not unexpected that we found no drastic changes in the function of the E1 protein or in the predicted structure.

From the evolutionary perspective, it appears that the E1-1374^∧^63nt E-G350 variant appeared among the HPV16 European lineage A2 sub-lineage variants, as it shares many variations with this sub-lineage (Figure 1). The initial events that occurred appear to be the addition of the 1053C and the 1374^∧^63nt changes within the E1 region, the GTTT insertion within the non-coding region at position 4211, and the 7450A change within the LCR. It is interesting that the A2 sub-lineage like variants containing the 4211 GTTT insertion and the LCR 7450A changes are common within the Indian population [15], thus the E1-1374^∧^63nt specific branching is likely to have initially occurred within such sub-lineage. Following this first evolutionary branching, the E1-1374^∧^63nt sub-lineage later split again with one branch acquiring additional LCR 24G and E6 109C changes. From the sequence analysis in this study, it appears that both sub-branches evolved separately and individually acquired further variations, however, as we found less variations within the 109C branch and this branch was present in fewer samples we can speculate that this 109C branch is more recent on the evolutionary scale. When considering our findings in the evolutionary context, we note that the usual A2 sub-lineage variants, historically represented by the E-G350 and the related variants, have never been previously associated with reduced cervical cancer risk or lower grade cervical lesions, but have been often associated with higher cancer risk [2]. However, after the E1-1374^∧^63nt branching, the association of the E1-1374^∧^63nt E-G350 with the lower grade cervical lesions was shown to be statistically significant, at least in comparison with the E-r variant and the E-G350 variants, the most common variants in the Croatian and many other populations worldwide [28]. From these phylogenetic considerations we can again conclude that changes within the E1 region might probably be the cause of the observed association.

The lack of obviously significant differences between different E1-1374^∧^63nt variants, E-G350 and E-C109/G350 was surprising. The only differences were the silent variations at position 24 C>G within the LCR and 109 T>C within the E6 region. To understand the possible significance of these variations, we reviewed the current literature searching for any potential effects these changes might have and found several possible mechanisms.

The LCR region has been extensively studied for binding sites of different cellular and viral factors important for HPV replication and transcription regulation (extensively reviewed in [54]–[57]). Position 24 itself is not within the binding site of any known transcription factors. However, it is located in the immediate vicinity of two potential binding sites, one for AP1 (nucleotides 15–21) and the other for SP1 (nucleotides 28–33). It is possible that, while the optimum consensus sequences of those transcription factors span only those mentioned positions, the transcription factor binding can be slightly influenced by a longer stretch of sequence.

The 109 T>C change is the signature variation of the E-C109/G350 variant. Position 109 is situated within the second codon of the 151 amino acid form of the E6 protein [58] but does not change the amino acid sequence of the E6 protein. This position is also interesting as it is in the immediate vicinity of the LCR region, which has always been investigated up to the promoter P97, although no sequence has thus far been analyzed for potential transcription factor binding sites after this position. However, the underlined sequence after the bolded start codon of the E6 protein, **ATG**·TTT·CA, corresponds to the AP1 factor binding site consensus (T[G/T]·[A/T]NT·[A/C]A) and almost perfectly to the predicted optimum sequence TG·ANT·CA (the only mismatch is underlined) [54]. We believe that this AP-1 site is actually capable of binding AP-1 transcription factor as the exact same sequence TG·TTT·CA is found in the ARRE-2 region of the human interleukin 2 promoter and is even conserved in birds [59]. In addition, the ARRE-2 site has been shown to be able to promote transcription when binding AP-1, even without other regulatory elements of the human interleukin 2 promoter [60]. The 109 T>C change corresponds to the sequence **ATG**·TTC·CA, which disrupts the consensus sequence that was found to accept only T in all oligonucleotides that bind AP1 at that changed position. Furthermore, it is known that HPV preferentially uses codons that are not optimal in its human host (reviewed in [61]). One of the reasons for this is probably to avoid the host immune response by reducing the production of viral proteins [62]. In this case, the 109 T>C substitution changes the phenylalanine codon TTT to the only other phenylalanine codon TTC. However, the TTT codon is preferentially used by the HPV (45.5/1000 codons) and not so by the human host (15.8/1000 codons), while the TTC codon is rarely used by HPV (only 4.0/1000 codons) but more commonly used by the human host (22.6/1000 codons) [63]. The confirmation that codon usage affects the HPV protein expression is the study by Cid-Arregui et al. [64] who showed that the codon optimized E7 protein is expressed 20–100 times more than the wild type HPV16 E7, and that the major difference was significantly improved translation. Another piece of evidence that the TTT codon might be suboptimal in E6 expression is from the study of Looman et al. [65], who show that it is the least optimal second codon of 31 possible second codons tested in a yeast expression system, with a 5.3 fold difference from best to worst. There are findings indicating that second codon can influence the efficiency of recognition of the start codon during translation. Phenylalanine that is situated at the second codon was rarely found in human proteins at that position [66], [67], suggesting that HPV might be using a suboptimal sequence to again reduce the protein levels in spite of strong enhancers [68]. That the second codon position is important, is also confirmed by the demonstration that codons at the 5' end of the mRNA can be rate limiting factors in protein synthesis [69] and can also influence premature translation termination [70]. In addition to directly repressing E6 protein translation, phenylalanine as the second codon could also be involved in E7 translation, as it appears that leaky scanning of E6/E7 bicistronic mRNA is the predominant mechanism of E7 translation [71]. The final possible mechanism involves translation frameshifting, as was shown in the study of Fu and Parker [72]. The authors showed that the sequence UUU·(U/C) can be misread by the ribosome so that instead of reading nucleotides 1 to 3 (UUU), nucleotides 2 to 4 are instead read UU·(U/C) as phenylalanine. In essence, this introduces a 1 bp deletion and a corresponding frameshift, which truncates the protein [72]. The frequency of such a frameshift was found to be 3–16% in the studied argI mRNA translation [72]. However, in the mutated sequence UUC·(U/C), this frameshift cannot take place. The same situation is found in the HPV16 sequence where UUU·C is changed in the E-C109 variant into UUC·C making the frameshift permissive sequence into an unpermissive sequence, again possibly influencing the E6 protein synthesis.

From all these studies it appears that the seemingly insignificant, silent change 109 T>C can very possibly affect the viral oncogene expression and its regulation, which probably influences viral life cycle and oncogenic potential of the E-C109/G350 and related variants. These direct changes to oncogene levels would very probably negate any effects of the E1 changes seen in the E1-1374^∧^63nt E-C109/G350 variant.

During the preparation of this manuscript, a report on E1-1374^∧^63nt variant was published by Bogovac et al. [8] from neighbouring Slovenia. The authors evaluated the prevalence of the variant containing the same 63 nucleotide duplication within the E1 region in 390 HPV16 positive cervical samples ranging from normal cytology to cervical cancer. The E1-1374^∧^63nt variant was found by RT-PCR in 31/390 (8%) samples, making its prevalence in Slovenia lower but still very close to the Croatian prevalence of this variant. Bogovac et al. [8] sequenced only the E6 region of the E1-1374^∧^63nt variant samples and only from nucleotide 273 to nucleotide 441 of the HPV16 genome to confirm the E-G350 association. Thus, it was not possible to compare our studies regarding the E1-1374^∧^63nt E-C109/G350 variant. The authors did see some trend of decreasing prevalence of E1-1374^∧^63nt variant from 14.6% in normal cytology samples to 7.1% in cancer samples, but without statistical significance. Bogovac et al. [8] confirmed their E1 RT-PCR results by sequencing and found the exact duplicated sequence within the E1 as we see in Croatian samples. However, as they sequenced only a 161 bp fragment of the E1 region (1258 to 1277) it is impossible to determine if their samples also contain any of the other E1 variations described in the current study.

In summary, we have found no significant changes in the whole genome of the E1-1374^∧^63nt E-G350 variant that differentiate this variant from the A2 sub-lineage variants, except in the E1 region. Thus, our findings indicate that E1 changes might significantly affect the virus, especially considering the essential role of the E1 protein in viral replication. However, further functional studies are required to elucidate the exact impact of these changes on the protein itself and its functions. The thorough research of the E1 gene variability, that was thus far neglected, definitely should be considered in future HPV16 variability studies. The current study indicates that variations in the E1 region might have an impact on the virus and therefore might affect the findings of studies investigating epidemiological association of variants and disease. This was most evident for the frequently studied 350 T>G variation, since the E1-1374^∧^63nt duplication is exclusively linked with this variation. The analysis of the E6 region alone, without consideration for the E1 region, could have confounded the results. Specifically, the exclusion of the E1 region analysis in the variability studies might be the cause for the current conflicting results, in the published literature, regarding the E-G350 variant and possibly the specific E-12 variant common in the Indian population. The unexpected finding in this study was the E1-1374^∧^63nt E-C109/G350 variant that was more associated with the high grade lesions than almost all other variants. From this study and the results found in the literature on the possible effects of the 109 T>C variation, it appears that this is not just an insignificant silent change but, on the contrary, affects several mechanisms of the viral life cycle. The E-C109/G350 variant definitely deserves more thorough study, especially in the light of its worldwide distribution and association with high grade cervical lesions.

## Supporting Information

Figure S1 **Prediction of E1 protein structure.** Panels A and C depict two different views of the 3D structure superposed comparison between a monomer of the previously solved BPV1 E1 helicase domain hexamer structure (cyan; PDB ID:2GXA) [25], HPV18 E1 helicase domain structure (yellow; PDB ID:1TUE) [26], BPV1 E1 DNA binding domain structure (red; PDB ID:1F08) [27] and the predicted referent E1 model (white). The sequence at the position where the duplication occurs within the E1-1374^∧^63nt variants is highlighted in green, as is the amino acid 63 that is also changed in the E1-1374^∧^63nt variants. Panels B and D depict two different views of the superposed structures of the referent (white) and the E1-1374^∧^63nt variant (magenta) E1 models. The referent sequence is highlighted in green and the changes specific to E1-1374^∧^63nt variant are highlighted in red. Panels E and F show two views of the E1 helicase domain hexamer structure (cyan; PDB ID:2GXA) with both E1 reference and E1-1374^∧^63nt variant structures superimposed on a single monomer of the solved structure.
(TIF)

Table S1 **Sequencing findings of the samples with unusual melting curves and the effect of specific variations on the 151 amino acid form of the E6 protein.**
(XLS)
