# Supplementary figures and images for: Characterization and Whole Genome Analysis of Human Papillomavirus Type 16 E1-1374∧63nt Variants
Source: PLoS One. 2012 Jul 24;7(7):e41045. doi: 10.1371/journal.pone.0041045 (PMC3404080; doi:10.1371/journal.pone.0041045)

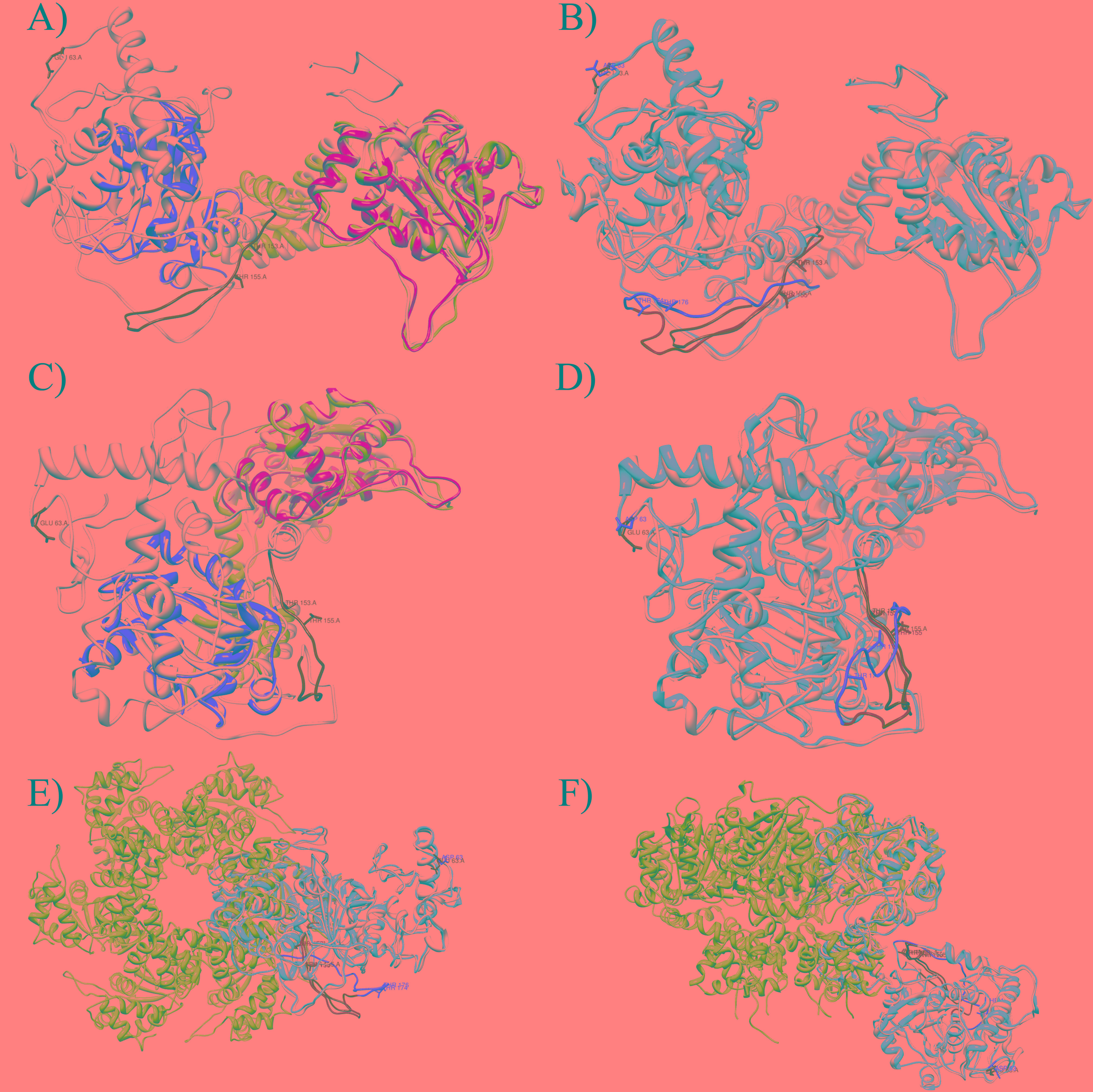

Supplement: Figure S1 — Prediction of E1 protein structure. Panels A and C depict two different views of the 3D structure superposed comparison between a monomer of the previously solved BPV1 E1 helicase domain hexamer structure (cyan; PDB ID:2GXA) [25], HPV18 E1 helicase domain structure (yellow; PDB ID:1TUE) [26], BPV1 E1 DNA binding domain structure (red; PDB ID:1F08) [27] and the predicted referent E1 model (white). The sequence at the position where the duplication occurs within the E1-1374∧63nt variants is highlighted in green, as is the amino acid 63 that is also changed in the E1-1374∧63nt variants. Panels B and D depict two different views of the superposed structures of the referent (white) and the E1-1374∧63nt variant (magenta) E1 models. The referent sequence is highlighted in green and the changes specific to E1-1374∧63nt variant are highlighted in red. Panels E and F show two views of the E1 helicase domain hexamer structure (cyan; PDB ID:2GXA) with both E1 reference and E1-1374∧63nt variant structures superimposed on a single monomer of the solved structure. (TIF) [file pone.0041045.s001.tif]
